# Effect of defatted winged termite (*Macrotermes natalensis*) meal on growth performance, blood metabolites and bone morphology of broiler chickens during starter, grower and finisher phases

**DOI:** 10.1016/j.psj.2026.106477

**Published:** 2026-01-18

**Authors:** Sekobane Daniel Kolobe, Amenda Nthabiseng Sebola, Emmanuel Malematja, Mabelebele Monnye

**Affiliations:** University of South Africa, Department of Agriculture and Animal Health, College of Agriculture and Environmental Sciences, Florida 1710, South Africa

**Keywords:** Broiler, Blood profile, Bone morphology, Growth performance, Termite meal

## Abstract

The current study was conducted to assess the effect of partial replacement of soyabean meal (SBM) (*Glycine max*) with defatted winged termite meal (WTM) (*Macrotermes natalensis*) inclusion levels on the performance, blood, and bone composition of Ross 308 broiler chickens in 42 days feeding trial. A total of 150 one-day-old, unsexed broiler chicks were allocated to 3 dietary treatments, replicated 5 times with 10 chickens per pen in a completely randomized design (CRD). Broilers were fed isonitrogenous, and iso-energetic experimental diets formulated to include WTM as follows: a control diet without WTM = WTM0; a basal broiler diet with 50g/kg of WTM = WTM5; a basal diet with 100g/kg of WTM = WTM10 to replace SBM. Average feed intake (FI), body weight gain (BWG) and feed conversion ratio (FCR) were measured at 1 to 14 days (starter), 15 to 28 days (grower) and 29 to 42 days (finisher). Hematology, serum biochemistry and right tibia bone traits of birds were determined on day 14, 28 and 42 in each growth phase. WTM inclusion had no effect (*P* > 0.05) on MR, BWG and FCR of broilers throughout the growth stages except FI that was higher (*P<* 0.05) in broilers fed WTM5 followed by WTM10 and WTM0 at starter phase. WTM inclusion had no (*P>* 0.05) influence on most hematology and serum parameters the growth period. However, elevated (*P<* 0.05) aspartate aminotransferase (AST) and bilirubin observed in chickens fed WTM5 than other groups at finisher phase. Dietary WTM inclusion had no influence (*P>* 0.05) on the majority of bone traits throughout the growth phase except bone density (TBD) that was higher (*P<* 0.05) in birds fed WTM5 than other treatment groups at starter phase whereas better (*P<* 0.05) bone breaking strength (BBS) was observed in birds on WTM5 compared to WTM0 and WTM10. It was concluded that WTM up to 10% could be partially included in diets without compromising growth performance, blood, and bone traits of Ross 308 broiler chickens during starter, grower and finisher phase.

## Introduction

The rapid growth of human population puts massive pressure on the livestock production industry worldwide. Moreover, demand for meat and meat products which is currently at about 60–70%, is expected to increase further by 2050 (Makkar et al., 2014). Poultry remains the topmost supplier of animal products globally due to its high nutritional value and affordability compared to beef and pork. In South Africa, chicken meat is considered a crucial meat source that helps curb hunger and food security, especially in poor communities (Oluwatayo et al., 2016). Thus, fast-growing chicken strains such as broilers remain an important commodity in the livestock farming industry in South Africa. However, broiler chicken farmers still struggle to maintain high productivity due to continuously rising costs of feed ingredients, mainly conventional protein sources (CPS) such as soybean and fish meal. Hence, the idea to substitute expensive feed ingredients with alternative feed sources of the same quality and quantity could bring relief to farmers such that they make more profit and improve their livelihood.

Edible insects such as black soldier flies and yellow meal worms are well documented as inexpensive alternative feed novel ingredient. They play an important role as dietary protein source for humans and livestock, especially in Africa and Asia (Egan et al., 2021; Dao et al., 2022). Recent studies observed that partial replacement of soybean meal (SBM) with the above-mentioned common commercial edible insects improved growth performance parameters, bone traits, nutrient digestibility and blood biochemistry in poultry species (Mutisya et al., 2022; Hamani et al., 2022; Yousif et al., 2022). Correspondingly, edible insects common to Southern Africa, such as termite alates, could potentially be a good alternative for CPS in poultry diets to improve productivity (Verner et al., 2021; Rumbos et al., 2020; Koutsos et al., 2023). Winged termites (*Macrotermes* spp.) are reproductive termites that mainly emerge during the early summer season after the first rains. Furthermore, unlike SBM (49% CP), they contain around 41 to 54% CP with essential fatty acids and minerals that are vital in improving the growth and performance of poultry species (Meyer-Rochow et al., 2021). However, only few studies reported on the inclusion of African termite species in poultry diets, especially local indigenous chickens (Pousga et al., 2019; Shindi et al., 2019; Susan et al., 2023; Tsukahara et al., 2023). Hence, there is limited information on the utilization of winged termites as a locally available and affordable alternative protein feed source in broiler chicken diets.

The potential farming and utilization of winged termites as animal feed could reduce food-feed competition of soybeans and its negative effect on the environment (Verner et al., 2021; Freccia et al., 2020; Tanga et al., 2021). However, edible insects contain are highly accumulated with chitin fibre, that binds to protein molecules and hinder its absorption and utilisation by chickens since they lack enzyme chitinase to breakdown chitin (Bonomini et al., 2024). Additionally, excessive fat content in edible insect significantly reduces protein solubility, quality and concentration (Marasca et al., 2025). Thus, processing of edible insects through prior to utilisation in livestock diets becomes critical (Fu et al., 2025). Hence, the objective of the current study was to determine the growth performance, blood and bone parameters of Ross 308 broiler chickens fed incremental levels of defatted winged termite meal (WTM) as a protein feed ingredient in diets. It was hypothesised that inclusion levels of WTM in diets had no effect on growth performance, blood metabolites and bone characteristics of Ross 308 broiler chickens at starter, grower and finisher phases.

## Material and methods

### Ethical statement and study site

The experiment was approved by the Animal Ethics Committee of the College of Agriculture and Environmental Science at the University of South Africa (UNISA), with approval number 2022/CAES_AREC/150.The study was carried out between August and September 2023 at the Izinkanyezi Chicken farm in Zuuberkom, Randfontein, South Africa. During that time the ambient temperature ranged between 14 to 29°C.

### Procurement of chicken and diet ingredients

Ross 308 broiler chicks (45 ± 0.4g) were purchased from National Chicks (Randburg, Gauteng, South Africa). Prior to experimental trials, three (3) iso-nitrogenous and iso-energetic treatment diets: 1) a basal diet without WTM =WTM0, 2) a basal diet containing 5% (50 g/kg) of WTM = WTM5 and 3) = a basal diet containing 10% (100 g/kg) of WTM = WTM10 to partially substitute soyabean meal (SBM) in broiler diets were formulated using Win feed 2.84 software. The defatted winged termite meal (WTM) along with other locally available feed ingredients including yellow maize, wheat and SBM (Table 1) were mixed, then crumbled or pelleted into 20kg bags at Simple Grow Agricultural Service, Johannesburg, South Africa.

**Table 1:** Ingredient and analysed chemical composition (%, unless stated otherwise) of experimental diets fed to broiler chickens aged during the starter, grower and finisher phases.

	4Diets (%)								
	Starter (1-14 days)			Grower (15-28 days)			Finisher (29-42 days)		
	WTM0	WTM5	WTM10	WTM0	WTM5	WTM10	WTM0	WTM5	WTM10
1Ingredients (%)									
Yellow maize fine	51.97	53.00	48.74	61.81	55.00	55.51	67.63	68.00	52.43
Pellibond (pellet binder)	1.00	1.00	1.00	1.00	1.00	1.00	1.00	1.00	1.00
Soy full-fat	8.99	-	-	-	-	-	-	-	-
Soya bean meal 46.5%	20.52	24.98	17.05	24.00	14.00	4.61	18.25	7.58	4.18
Sunflower oilcake 34%	10.79	3.00	2.00	6.00	8.21	10.05	5.61	10.04	-
Termite meal	-	5.00	10.00	-	5.00	10.00	-	5.00	10.00
Wheat bran	-	7.62	17.05	-	10.47	15.00	-	3.37	25.52
Crude soya oil	3.00	1.49	-	3.51	2.80	-	4.50	1.76	3.39
L-lysine HCL	0.47	0.44	0.56	0.44	0.59	0.74	0.49	0.60	0.63
DL-Methionine	0.17	0.18	0.20	0.18	0.19	0.20	0.16	0.16	0.22
L-Threonine	0.20	0.21	0.27	0.15	0.21	0.27	0.14	0.18	0.25
L- Tryptophan	-	0.01	0.03	-	0.02	0.05	-	0.03	0.04
L-Valine	0.09	0.07	0.14	-	-	-	0.08	0.14	0.22
Feed lime fine	1.17	1.36	1.36	1.15	1.22	1.23	1.05	1.03	1.06
MDCP	0.88	0.92	0.94	0.78	0.62	0.62	0.45	0.50	0.44
Salt fine	0.20	0.20	0.20	0.27	0.21	0.15	0.20	0.20	0.18
Sodium bicarbonate	0.14	0.11	0.08	0.25	0.10	0.20	0.15	0.12	0.12
Choline chloride 60% powder	-	-	-	0.11	-	-	-	-	-
Avatec 15% (coccidiostat)	0.05	0.05	0.05	0.05	0.05	0.05	0.05	0.05	0.05
Zinc bacitracin 15% (AGP)	0.05	0.05	0.05	0.05	0.05	0.05	0.05	0.05	0.05
AxtraPhy broiler phytase	0.01	0.01	0.01	0.01	0.01	0.01	0.01	0.01	0.01
Broiler finisher Premix	0.30	0.30	0.30	0.25	0.25	0.25	0.20	0.20	0.20
Total	100	100	100	100	100	100	100	100	100
2Analysed nutrient composition (%)									
DM	89.07	89.03	88.59	89.18	88.84	89.31	89.34	89.11	89.48
Ash	4.57	5.21	5.62	4.47	4.77	4.92	3.71	4.15	4.42
CP	21.91	22.60	22.41	19.75	19.68	20.07	17.35	17.57	18.04
CF	4.13	4.55	3.27	3.69	4.54	6.39	5.04	5.54	5.83
EE	6.70	5.85	5.54	6.60	6.91	5.09	7.25	6.34	8.74
ME(MJ/kg)	12.55	12.10	12.81	12.11	12.69	12.33	12.04	12.11	12.94
**Minerals**									
Calcium	0.90	0.96	0.96	0.84	0.84	0.84	0.72	0.72	0.72
Phosphorus	0.45	0.45	0.45	0.72	0.74	0.76	0.62	0.66	0.71
Magnesium	0.19	0.16	0.16	0.16	0.17	0.17	0.14	0.15	0.14
Potassium	0.95	0.88	0.80	0.84	0.76	0.64	0.72	0.60	0.60
Sodium	0.16	0.16	0.16	0.21	0.16	0.17	0.16	0.16	0.16
Chlorine	0.26	0.26	0.29	0.30	0.30	0.30	0.26	0.29	0.30
Sulphur	-	-	-	0.02	0.02	0.02	0.02	0.21	0.01
3Amino acids									
Lysine	1.26	1.22	1.22	1.12	1.12	1.12	1.02	0.97	0.97
Methionine	0.48	0.46	0.46	0.45	0.45	0.45	0.40	0.40	0.41
Cystine	0.29	0.27	0.25	0.26	0.24	0.22	0.23	0.21	0.19
Met + Cys	0.77	0.73	0.71	0.71	0.69	0.67	0.63	0.61	0.60
Threonine	0.86	0.83	0.83	0.73	0.73	0.73	0.64	0.62	0.62
Tryptophan	0.21	0.20	0.20	0.18	0.18	0.18	0.15	0.15	0.15
Isoleucine	0.79	0.72	0.63	0.69	0.61	0.50	0.59	0.51	0.41
Arginine	1.31	1.24	1.18	1.11	1.07	1.01	0.94	0.91	0.82
Phenylalanine	0.90	0.84	0.74	0.81	0.71	0.60	0.69	0.61	0.49
Histidine	0.47	0.46	0.42	0.43	0.39	0.34	0.38	0.33	0.29
Leucine	1.56	1.49	1.34	1.44	1.28	1.15	1.30	1.17	1.10
Tyrosine	0.62	0.59	0.52	0.56	0.48	0.41	0.48	0.41	0.34
Valine	0.96	0.89	0.89	-	-	-	0.74	0.74	0.74
Alanine	-	-	-	-	-	-	0.71	0.75	0.76
Aspartic acid	-	-	-	-	-	-	1.31	1.07	0.85
Glutamine	-	-	-	-	-	-	2.57	2.32	2.01
Glycine	0.73	0.91	1.10	-	-	-	0.54	0.75	0.92
Proline	-	-	-	-	-	-	0.88	0.91	0.95
Serine	-	-	-	-	-	-	0.71	0.60	0.53
Gly + Ser	-	-	-	-	-	-	1.28	1.41	1.50

1 Ingredients: MDCP = Monodicalcium phosphate

2 Analysed composition: DM = Dry matter, CP= Crude protein, CF= Crude fibre, EE= Ether extract, ME= Metabolizable energy.

3 Amino acids: Met + Cys = Methionine + Cystine; Gly + Ser = Glycine + Serine

4 Diets: WTM0 = broiler diet without winged termite meal; WTM5 = broiler chicken diet containing 5% of winged termite meal; WTM10 = broiler chicken diet containing 10% of winged termite meal; ‘-‘= not incorporated.

### Preparation and management of chicken house

The experimental house and its equipment were thoroughly cleaned and disinfected prior to feeding trials. Thereafter, house was divided into 15 wire-mesh floor pens (experimental units) of equal sizes (100 cm long × 60 cm wide × 30 cm high). Fresh wood shavings were spread on the floor to a thickness of 5 cm deep as bedding for birds. The chicks were then brooded at 32-33°C for the first day and then dropped by 1°C for every 3 days till room temperature. Fresh, clean water and diets were offered *ad libitum* during the 42-day feeding trial. The chicks were offered a stress pack (containing vitamins and electrolytes) administered through drinking water for the first three days of the trial. Lights were provided 20 h (9 h of natural light and 11 hours of bulb or artificial light) daily and maintained throughout the trial period with 4 h of darkness. Mortality was recorded instantly and thereafter; a post-mortem was conducted by a registered veterinarian to determine the cause of death. The dead birds were subsequently incinerated in line with the UNISA Animal Research Ethics Committee regulations.

### Experimental design and treatment diets

A total 150 unsexed Ross 308 broilers day-old chicks were assigned 3 treatments in a completely randomized design (CRD) replicated five (5) times with ten (10) birds per replica (pen). Each diet plan (WTM0, WTM5 and WTM10) created using Win Feed 2.84 software was formulated based on the chemical composition of the ingredients used in order to meet or exceed the nutritional requirements of Ross broiler chicken (Aviagen, 2014) starter (1 to 14 days), grower (15 to 28 days), and finisher (29 to 42 days) diets. The feed ingredient and chemical composition as well as the proximate composition of WTM are shown in Table 1, Table 2, respectively.

**Table 2:** Proximate composition (%) of WTM (*M. natalensis*) used in feed formulation.

1**Parameters**	**WTM**
Dry matter	95.60
Crude protein	53.64
Crude fat	22.51
Crude fibre	6.83
NDF	26.50
Ash	9.67

1 Parameters: NDF = Neutral detergent fibre. ^2^WTM: Winged termites meal, a by-product of *M. natalensis*

### Growth performance

#### Mortality rate, average daily gain and body weight gain

Chicken mortality (%MR) in each pen was recorded as and when it occurs and was calculated using Formula 1. The initial and final weight were measured using a digital weighing scale (Adam Aqua ABW4 scale, South Africa). Thereafter, the average daily gain was calculated using Formula 2, by subtracting the initial live weight of the bird from the final live weight and divided by the number of days.

(1) $$\text{Percentage}\;\text{mortality}\;\text{rate}\;\left(\text{MR}\right)=\frac{\text{Total}\;\text{number}\;\text{of}\;\text{dead}\;\text{birds}}{\text{Total}\;\text{number}\;\text{of}\;\text{chickens}\;}x100$$

(2) $$\text{Average}\;\text{daily}\;\text{gain}\;\left(\text{ADG}\right)\;\left(g/\;\text{bird}/\text{day}\right)=\frac{\text{Final}\;\text{weight}-\text{Initial}\;\text{weight}\;}{\text{number}\;\text{of}\;\text{trial}\;\text{days}\;}$$

#### Feed intake (FI)

Feed offered and residuals were weighed daily using the electronic weighing balance (Ebal WS-30 30kg x 1g) such that FI using Formula 3.

(3) $$\text{Feed}\;\text{intake}\;\left(\text{FI}\right)\;\left(g/\;\text{bird}/\text{day}\right)=\frac{\text{Feed}\;\text{offered}\;--\;\text{Feed}\;\text{residuals}}{Total\;number\;of\;birds\;in\;a\;pen}$$

#### Feed conversion ratio (FCR)

The FCR per bird was calculated as the total amount of feed intake (FI) divided by average daily gain (ADG) of the chicken as shown in Formula 4 below.

(4) $$\text{FC}=R\;\frac{\text{Feed}\;\text{intake}}{Average\;daily\;gain\;}$$

### Blood profiles

#### Hematology

Blood samples were collected at 14, 28 and 42 days from two randomly selected birds in each replicate or pen to determine the hematological indices. About 2 ml of blood was drawn from the wing vein of each bird using a 21-gauge needle, which was then injected into the purple top 4 ml ethylene diamine tetra-acetic acid (EDTA)-coated vacutainer tubes for the determination of haematological parameters, including white blood cell count (WBC), heterophils, heterophils, lymphocytes, monocytes, eosinophils, basophils and the packed cell volume (% PCV) were determined using an automated LaserCyte. The tubes containing the blood were carefully stored in a cooler bag with ice cubes and immediately transported to a Veterinary Hospital at the University of Pretoria. The haematology samples were analysed within 24 hours after collection. Moreover, the mean ratio of the heterophils to lymphocytes ratio was also calculated by dividing the number of heterophils with lymphocytes cells as described by Thiam et al*.,* (2022).

### Serum analysis

The collected blood in a red top vacutainer tubes without EDTA was used to determine serum biochemistry indices including total serum protein (TSP), albumin, alanine aminotransferase (ALT), urea, aspartate aminotransferase (AST), cholesterol, amylase, triglycerides, gamma-glutamyl transpeptidase (GGT), uric acid, bilirubin, serum sodium, potassium, chloride, calcium, glucose, iron and magnesium which were analysed using an automated Vet Test Chemistry Analyser (IDEXX Laboratories (Pty) Ltd. Johannesburg, South Africa).

### Determination of bone measurements

On days 14, 28 and 42, two (2) birds from each pen were randomly selected, weighed (Ebal WS-30 30kgx1g), and humanely killed by cervical dislocation method. Immediately after slaughter, the right tibia bones were harvested and then measured for tibia bone weight (TBW), length (TBL) and diameter (BD) using a digital calliper (Vanier calliper) set in millimetres (mm). The tibia bone density (TBD), on the other hand was calculated according to formula 5 below. The tibiae were individually sealed in plastic bags to minimise moisture loss and stored in a freezer at -18°C for later analysis. TBD was calculated by dividing the tibia weight (g) by the tibia length (mm) as shown in formula 5 below. Tibia bone breaking strength (BBS) was determined by placing the bone horizontally between brackets set at 10 mm apart. The breaking strength was measured by applying pressure using a Texture analyser machine (TA XT plus, Stable Micro System, Surrey, UK).

(5) $$\text{Tibia}\;\text{bone}\;\text{density}\;\left(\text{TBD}\right)=\frac{Tibia\;weight\;\left(g\right)}{Tibia\;length\;\left(mm\right)}$$

### Statistical analysis

The MR, FI, ADG, FCR, haematology and serum biochemistry data were analysed using one-way analysis of variance from general linear model (GLM) using Statistical Analysis System (SAS, 2019) according to the following model: Y_ij_ = μ + d_i_ + E_ij_. Where Y_ij_ = response variables (MR, FI, ADG, FCR, haematology and serum biochemistry indices), µ = general mean, d_i_ = the fixed effects of diets, E_ij_ = random error associated with observation ij = assumed to be normally and independently distributed. Where there are significant differences among treatment means, the Tukey’s honestly significant difference (HSD) option was used to separate means. The level of significance was set at (*P* *<* 0.05).

## Results

### Growth performance parameters

The effect of defatted WTM inclusion levels on MR, FI, BWG and FCR of Ross 308 broiler chicken diets at starter, grower and finisher phase is represented in Table 3. Throughout the growing period, WTM did not affect (*P* *>* 0.05) MR, BWG and FCR of broiler chickens with exception of FI that was significantly affected during starter phase. Higher (*P* *<* 0.05) FI was observed at starter phase on broiler chicks fed diet WTM5 compared to those on WTM10 and WTM0, respectively. However, broiler chicks on WTM10 had similar (*P* *>* 0.05) FI with those fed WTM5 and WTM0 diets. FI of broiler chicks on WTM at grower and finisher phases were not affected (*P* *>* 0.05) by the experimental diets.

**Table 3:** Effect of WTM inclusion levels on % mortality rate (MR), feed intake (FI) (As fed), average daily gain (ADG) and feed conversion ratio (FCR) (of Ross 308 broiler chickens at starter (1 to 14 days), grower (15 to 28 days) and finisher (29 to 42 days) phase.

1Parameters	Inclusion level (%) *			2SEM	P-value
	WTM0	WTM5	WTM10		
**MR** (%)					
Starter	0.00	0.00	0.20	0.62	0.08
Grower	0.60	0.40	0.60	0.91	0.12
Finisher	0.20	0.40	0.40	0.21	0.32
**FI (g/bird/day)**					
Starter	420.55^b^	464.09^a^	442.38^ab^	9.42	0.02
Grower	805.28	846.86	824.91	45.93	0.82
Finisher	630.90	647.50	556.55	36.18	0.21
**BWG (g/bird/day)**					
Starter	464.00	483.48	465.03	12.71	0.65
Grower	1687.53	1636.27	1593.40	40.29	0.52
Finisher	2711.50	2663.30	2402.70	36.65	0.56
**FCR (g feed/g live weight gain)**					
Starter	1.30	1.45	1.31	0.02	0.15
Grower	1.27	1.48	1.42	0.09	0.31
Finisher	1.41	1.56	1.40	0.14	0.67

1 Parameters: FCR = Feed Conversion Ratio

2 SEM = Standard Error of Mean ^a, b, c^ = Means with different superscripts in the same row indicate significant differences between treatments (p<0.05). * Inclusion levels: WTM0 = broiler diet without winged termite meal; WTM5 = broiler chicken diet containing 5% of winged termite meal; WTM10 = broiler chicken diet containing 10% of winged termite meal.

### Hematology

The results on the effect of partial inclusion of WTM on hematology indices, including white blood cell count (WBC), heterophils, lymphocytes, monocytes, eosinophils, basophils, packed cell volume (%PCV) and heterophil to lymphocytes (H/L) ratio of broiler chicken at the starter, grower and finisher phase are represented in Table 4. WTM diets had no effect on (*P* *>* 0.05) heterophils, monocytes, eosinophils and basophils throughout the growth period except WBC, lymphocytes, % PCV and H/L ratio which were significantly affected only at grower phase. Birds on WTM10 had higher (*P* *<* 0.05) WBC and lymphocyte levels than those on WTM5 and WTM0, respectively. However, broilers having WTM5 had similar (*P* *>* 0.05) WBC levels than those on WTM0 and WTM10 diets. Similarly, lymphocytes levels of birds on WTM5 and WTM0 were the same (*P* *>* 0.05). Higher (*P* *<* 0.05) % PCV was observed in broilers on WTM5 and WTM10 than those fed control diet. Broilers on WTM10 had lower (*P* *<* 0.05) H/L ratio than those having diets WTM0 and WTM5, which were the same (*P* *>* 0.05).

**Table 4:** Haematological parameters in Ross 308 broiler chickens fed incremental levels of WTM during the starter, grower and finisher phases.

1Parameters	Inclusion level (%)*			2SEM	P-value
	WTM0	WTM5	WTM10		
**WBC** (x10^9^/L)					
Starter	5.95	5.23	5.00	1.20	0.85
Grower	5.73^b^	7.02^ab^	8.86^a^	0.59	0.03
Finisher	7.82	7.71	7.36	1.11	0.95
**Heterophil** (x10^9^/L)					
Starter	2.88	3.08	2.96	0.49	0.96
Grower	3.91	4.93	4.27	0.27	0.09
Finisher	3.24	3.77	4.14	0.89	0.78
**Band Heterophil** (x10^9^/L)					
Starter	0.21	0.15	0.00	0.05	0.09
Grower	0.27	0.03	0.10	0.08	0.17
Finisher	0.15	0.07	0.09	0.06	0.62
**Lymphocytes** (x10^9^/L)					
Starter	1.19	1.10	0.89	0.44	0.89
Grower	0.97^b^	0.91^b^	3.12^a^	0.26	<0.01
Finisher	2.38	1.83	1.89	0.50	0.71
**Monocytes** (x10^9^/L)					
Starter	0.89	0.35	0.47	0.37	0.58
Grower	0.20	0.51	0.61	0.16	0.23
Finisher	0.31	0.79	0.93	0.31	0.36
**Eosinophils** (x10^9^/L)					
Starter	0.35	0.21	0.51	0.12	0.25
Grower	0.27	0.25	0.20	0.07	0.81
Finisher	0.98	0.84	0.24	0.22	0.11
**Basophils** (x10^9^/L)					
Starter	0.47	0.36	0.17	0.17	0.52
Grower	0.36	0.23	0.70	0.17	0.21
Finisher	0.75	0.29	0.06	0.21	0.12
**PCV** (%)					
Starter	30.33	30	29.67	1.62	0.96
Grower	30.33^b^	35.67^a^	36.00^a^	0.64	<0.01
Finisher	32.33	32.67	36.33	1.55	0.09
**H/L ratio**					
Starter	3.36	4.26	3.37	1.34	0.87
Grower	4.95^a^	5.42^a^	1.39^b^	0.81	0.03
Finisher	1.88	2.11	2.71	0.85	0.71

1 Parameters: WBC = White blood cell count; PCV = Packed cell volume; H/L ratio = Heterophil to Lymphocytes ratio

2 SEM = Standard Error of Mean ^a, b, c^: Means with different superscripts in the same row indicate significant differences between treatments (p < 0.05). * Inclusion levels: WTM0 = broiler diet without winged termite meal; WTM5 = broiler chicken diet containing 5% of winged termite meal; WTM10 = broiler chicken diet containing 10% of winged termite meal.

### Serum biochemistry

The effect of WTM inclusion levels on serum biochemistry indices, including total serum protein (TSP), albumin, alanine aminotransferase (ALT), aspartate aminotransferase (AST), cholesterol, amylase, triglycerides, gamma-glutamyl transpeptidase (GGT), uric acid, bilirubin and glucose, as well as serum minerals (sodium, urea, potassium, chloride, calcium, iron and magnesium) of broiler chicken at the starter, grower and finisher phase are showed in both Table 5, Table 6. The incremental levels of WTM did not affect (*P* *>* 0.05) TSP, cholesterol, amylase, triglycerides, GGT, uric acid, bilirubin glucose, sodium, urea, potassium, chloride, calcium, iron and magnesium throughout the growing period with exception of ALT, AST and bilirubin that were affected (*P* *<* 0.05) by WTM inclusion in diets at finisher phase only. Higher (*P* *<* 0.05) ALT was observed in chickens fed WTM5 followed by WTM0 and WTM10. However, chickens on WTM0 diet had similar (*P* *>* 0.05) ALT concentration with those on WTM5 and WTM10 diets. AST and bilirubin levels of birds having WTM5 diet were higher (*P* *<* 0.05) than those fed WTM0 and WTM10, which were similar (*P* *>* 0.05).

**Table 5:** Serum parameters in Ross 308 broiler chickens fed incremental levels of WTM during the starter, grower and finisher phases.

1Parameters	Inclusion level (%) *			2SEM	P-value
	WTM0	WTM5	WTM10		
**TSP (g/l)**					
Starter	22.80	29.73	22.40	2.65	0.17
Grower	25.33	28.00	29.40	1.13	0.10
Finisher	30.17	31.27	25.87	2.91	0.43
**Albumin (g/l)**					
Starter	11.80	12.37	9.43	1.16	0.24
Grower	12.63	14.97	14.10	1.03	0.34
Finisher	17.47	14.10	13.77	1.72	0.31
**ALT (U/L)**					
Starter	1.83	11.00	1.07	3.34	0.14
Grower	1.17	1.97	2.40	1.60	0.86
Finisher	1.40^ab^	3.47^a^	0.07^b^	0.73	0.04
**AST (U/L)**					
Starter	197.43	313.03	184.77	48.41	0.21
Grower	233.77	432.80	332.66	73.19	0.24
Finisher	291.03^b^	511.20^a^	267.30^b^	23.48	<0.01
**Cholesterol (mmol/l)**					
Starter	3.01	3.27	2.88	0.16	0.30
Grower	3.08	3.15	3.04	0.36	0.98
Finisher	3.40	2.87	3.03	0.26	0.39
**Amylase (U/L)**					
Starter	698.97	748.67	678.03	124.68	0.92
Grower	657.63	768.40	612.10	96.71	0.54
Finisher	481.80	520.43	620.30	100.37	0.63
**Triglycerides (mmol/l)**					
Starter	0.80	1.17	0.75	0.12	0.09
Grower	0.66	0.77	0.84	0.17	0.77
Finisher	0.67	0.53	1.04	0.32	0.55
**GGT (U/L)**					
Starter	5.23	24.63	9.90	5.02	0.08
Grower	14.23	18.07	17.73	3.42	0.71
Finisher	16.47	12.40	19.43	2.36	0.19
**Uric acid (mmol/l)**					
Starter	0.26	0.34	0.41	0.06	0.29
Grower	0.20	0.24	0.17	0.04	0.48
Finisher	0.24	0.19	0.18	0.04	0.51
**Bilirubin (mmol/l)**					
Starter	0.20	0.03	0.03	0.07	0.25
Grower	0.07	0.07	0.17	0.09	0.67
Finisher	0.03^b^	0.70^a^	0.00^b^	0.12	0.01
**Glucose (mmol/l)**					
Starter	16.89	15.41	16.77	0.82	0.42
Grower	14.23	14.82	14.61	0.41	0.62
Finisher	13.31	12.82	14.61	0.45	0.07

1 Parameters: TSP = Total serum protein; ALT = Alanine aminotransferase; AST = Aspartate aminotransferase; GGT = Gamma-glutamyl transpeptidase.

2 SEM = Standard Error of Mean ^a, b, c^: Means with different superscripts in the same row indicate significant differences between treatments (p < 0.05). * Inclusion levels: WTM0 = broiler diet without winged termite meal; WTM5 = broiler chicken diet containing 5% of winged termite meal; WTM10 = broiler chicken diet containing 10% of winged termite meal.

**Table 6:** Serum parameters (mmol/l) in Ross 308 broiler chickens fed incremental levels of WTM during the starter, grower and finisher phases (continuation of Table 5).

Parameters	Inclusion level (%) *			1SEM	P-value
	WTM0	WTM5	WTM10		
**Sodium**					
Starter	144.10	142.17	146.00	1.22	0.17
Grower	147.97	148.53	147.17	1.05	0.67
Finisher	150.23	144.80	147.07	3.45	0.57
**Urea**					
Starter	0.27	0.48	0.25	0.13	0.44
Grower	0.17	0.37	0.22	0.09	0.34
Finisher	0.22	0.28	0.17	0.04	0.21
**Potassium**					
Starter	6.43	10.39	6.83	1.52	0.21
Grower	5.97	6.01	6.53	0.58	0.76
Finisher	6.49	6.25	6.64	0.48	0.85
**Chloride**					
Starter	109.87	108.00	111.43	1.26	0.23
Grower	12.83	113.80	113.37	1.15	0.84
Finisher	115.00	111.07	109.87	2.23	0.31
**Calcium**					
Starter	2.57	2.21	2.51	0.14	0.22
Grower	2.58	2.63	2.64	0.05	0.74
Finisher	2.37	2.14	2.35	0.08	0.15
**Iron**					
Starter	16.03	19.77	18.77	1.70	0.34
Grower	12.57	20.43	18.63	2.66	0.17
Finisher	16.77	19.63	17.53	2.61	0.74
**Magnesium**					
Starter	0.91	1.04	0.99	0.07	0.41
Grower	0.92	0.95	0.88	0.04	0.50
Finisher	0.82	0.78	0.83	0.05	0.68

1 SEM = Standard Error of Mean ^a, b, c^: Means with different superscripts in the same row indicate significant differences between treatments (P<0.05). * Inclusion levels: WTM0 = broiler diet without winged termite meal; WTM5 = broiler chicken diet containing 5% of winged termite meal; WTM10 = broiler chicken diet containing 10% of winged termite meal.

### Bone measurements

The results on the effect of WTM inclusion levels on tibia bone characteristics, including TBL, TBW, TBD, BD and BBS of broilers during the starter, grower and finisher phases are presented in Table 7. WTM inclusion had no effect (*P* *>* 0.05) on TBL, BD and BBS at the starter, TBL and BD at the grower, and TBL, TBW, BD, TBD and BBS at the finisher phase. However, chicken TBW and TBD at the starter and TBW, TBD and BBS at the grower phase were significantly (*P* *<* 0.05) affected by WTM inclusion in diets. At the starter phase, higher (*P* *<* 0.05) TBW and TBD were found in broilers fed WTM5 than those on WTM0 or WTM10, which were similar (*P* *>* 0.05) in TBW and TBD. However, broilers on WTM0 and WTM5 also had the same (*P* *>* 0.05) TBW. At the grower period, chickens on the control diet had higher (*P* *<* 0.05) TBW and TBD than those on WTM5 or WTM10. Moreover, broilers on WTM0 and WTM5 also had similar (*P* *>* 0.05) TBW and TBD. Higher (*P* *<* 0.05) BBS was observed in birds having WTM5 than those fed WTM0 or WTM10, which had similar (*P* *>* 0.05) BBS.

**Table 7:** Effects of replacing SBM with WTM on right tibia characteristics of broiler chickens at the starter (1 to 14 days), grower (15 to 28 days) and finisher (29 to 42 days) phases.

1Tibia characteristics	Inclusion level (%)*			^2^SEM	P-value
	WTM0	WTM5	WTM10		
**TBL (mm)**					
Starter	56.17	53.33	52.83	1.71	0.39
Grower	80.00	78.17	77.83	0.95	0.31
Finisher	81.33	79.33	79.00	1.19	0.38
**TBW (g)**					
Starter	4.26^ab^	4.67^a^	4.05^b^	0.12	0.03
Grower	5.61^a^	4.45^ab^	4.08^b^	0.29	0.03
Finisher	7.32	6.74	5.55	0.43	0.07
**BD (mm)**					
Starter	4.50	4.50	4.50	0.24	1.00
Grower	6.33	6.25	5.67	0.33	0.36
Finisher	6.33	6.50	5.83	0.27	0.27
**TBD (gmm^−1^)**					
Starter	0.08^b^	0.09^a^	0.08^b^	<0.01	0.01
Grower	0.07^a^	0.06^ab^	0.05^b^	<0.01	0.03
Finisher	0.09	0.09	0.07	0.01	0.12
**BBS (N)**					
Starter	413.40	344.20	340.30	92.24	0.83
Grower	376.43^b^	528.77^a^	373.07^b^	30.87	0.02
Finisher	338.00	487.70	248.30	73.54	0.15

1 Tibia parameters; TBL = tibia bone length, TBW = tibia bone weight, BD = bone diameter, TBD = tibia bone density, BBS = bone breaking strength. ^2^SEM = Standard Error of Mean ^a, b^: Means with different superscripts in the same column indicate significant differences between treatments (p < 0.05). * Inclusion levels: WTM0 = broiler diet without winged termite meal; WTM5 = broiler chicken diet containing 5% of winged termite meal; WTM10 = broiler chicken diet containing 10% of winged termite meal.

## Discussion

### Growth performance

The WTM inclusion non-significant effects on performance parameters including MR, FI, BWG and FCR of broiler chickens throughout the growing period. Similar observations were made by Shindi et al., (2019) and Ke et al., (2017) when including termites in broiler diets. Susan et al. (2023) observed that termite inclusion levels at 0, 2.5 and 5% in indigenous chicken diets had no effect on FCR. Furthermore, another study also used similar inclusion levels (0.5, 10 and 15%) and concluded that growth parameters did not vary significantly after supplementing yellow mealworm as a replacement for SBM (Ramos-Elorduy et al., 2002). This suggests that WTM could be partially included in broiler chicken diets without adversely affecting the overall performance. However, the results are contrary to that of Amobi and Ebenebe, (2018a,b), who reported significant differences in BWG of broilers fed control and winged termite diets. This contradiction may be due to the incorporation of different edible insect species and varying inclusion levels in chicken diets.

Nonetheless, elevated FI of chicks fed WTM diets than control group during starter phase aligns with reports by Amobi and Ebenebe, (2018a,b). A possible explanation of could be better taste, flavour and palatability of termites when supplemented in poultry diets (Sogbesan and Ugwumba, 2008; Vugutsa et al., 2022; Kinyuru, 2014). Moreover, defatting of WTM prior to incorporation in diets may have improved its utilization by chicks since processing techniques, particularly oil extraction is known to improve nutrient availability. This is in line with reports made by Mali et al., (2020) and Deori et al., (2022). However, there is limited information on the digestibility and utilization of WTM by broiler chickens. Hence, further research on digestibility protein intake and efficiency of WTM is needed to ascertain the partial replacement of SBM with WTM in broiler diets.

### Hematology parameters

Hematology involves the vital diagnosis of blood from a tissue and cell level to determine any abnormalities, diseases and pathogens that might interfere with normal health of the animal in response to the quality of diet provided (Adekunle and Omoh, 2014). This includes examining the fluidity, circulation, and clotting ability of blood within a certain period (Tijani et al., 2015). The partial inclusion of WTM as a protein source in broiler diets had no effect on all blood hematology traits, with exception of WBC, lymphocytes, %PCV and H/L ratio only at grower phase. Although there was slight elevation of WBC and %PCV in birds on WTM diets as well as increased lymphocytes levels in birds on WTM10 compared to control group at grower phase, their values still fell within the acceptable range, as noted by Etim et al., (2014). Thus, indicating that the inclusion of WTM in diets did not tamper with the immune system status of broiler chickens considering that blood hematology parameters highly sensitive to diet modifications (Adekunle and Omoh, 2014). Similar reports were made by Dabbou et al., (2018). However, the slight increase in WBC and %PCV in WTM diets could be due to the presence of high dietary fibre (chitin) in WTM diets (Table 1), which has been reported to modulate the immune system response in broiler chickens (Mohassesi et al., 2025). The H/L ratio which is mainly used for measuring distress conditions in birds (Dabbou et al., 2018), was lower in broilers fed WTM10 compared to other treatment groups during the grower phase. Indicating that birds did not suffer from any infections, inflammation, or stress. This in agreement with findings made by Thiam et al., (2022). According to Bovera et al. (2015) and Elahi et al. (2022), chitin compounds found in insect meals at high levels are responsible for promoting better disease resistance and immune response by lowering H/L ratio in chickens, thus improving birds health.

### Serum biochemical indices

Serum parameters not only determine the nutritional status within the blood but also play a significant role in viscosity and balancing both blood pressure and pH (Adekunle and Omoh, 2014). It is the watery portion of blood containing proteins as well as antibodies, electrolytes, antigens, and hormones, which are vital substances that aid in the normal functioning of the animal, such as fighting against diseases in animals (Tijani et al., 2015). In birds, the quality of diet provided to the animal could particularly influence their serum biochemical parameters.

The non-significant effect inclusion of WTM on the majority of chicken serum parameters, including total serum protein (TSP), albumin, cholesterol, amylase, triglycerides, GGT, uric acid, glucose, sodium, urea, potassium, chloride, calcium, iron and magnesium throughout the growth period implies that WTM could be included in broiler diets without tempering with blood serum biochemistry. The significant findings in alanine aminotransferase (ALT), aspartate aminotransferase (AST) and bilirubin observed by Dabbou et al. (2018) and Bovera et al. (2015) after supplementing 0, 5, 10 and 15% of BSF in broiler diets at finisher phase are in line with the results of the current study.

ALT and AST are vital enzymes secreted in large quantities in the blood when there is liver damage and distress suffered by birds (Ognik and Krauze, 2016) while bilirubin plays a role in maintaining antioxidant balance in birds and thus regarded as a vital physiological indicator of antioxidant status (Capcarová et al., 2013). Bilirubin is also responsible for influencing changes in metabolism, immunity, and vascular function of the animal (Vitek et al., 2023). The current results indicates that of ALT enzymes were significantly higher and lower in broilers fed WTM5 and WTM10, respectively at finisher phase. Additionally, higher levels of AST and bilirubin in chickens fed WTM5 than other dietary groups during the finisher. Although lower levels of ALT indicate a good health status of the liver while higher ALT, AST and bilirubin is associated with liver damage (Upah et al., 2024), values of ALT, AST and bilirubin observed in the current study still fell within the normal physiological range. Thus, indicating that WTM diets did not affect the normal functioning of the liver, cause any nutritional-related stress (Adekunle and Omoh, 2014; Bovera et al., 2015). Nonetheless, the results are contrary to finding by Hatab et al., (2020) who observed non significance in ALT, AST and bilirubin when up to 100% insect meal was included in quail diets. The existing contradiction may be due to varying inclusion levels and poultry species.

### Bone morphometrics

The susceptibility of modern broiler chickens to bone abnormalities due to rapid growth rates has been documented (Shim et al., 2012). Although there was absent variation in most of the tibia bone traits throughout the growth period, TBD at starter, and TBW, TBD and BBS at finisher phase varied significantly. Slight elevation in TBW and TBD of broilers on WTM5 compared to WTM10 at starter phase may be due to the protein and mineral contents in WTM diets, which tended to promote bone growth and density. This could be linked with elevated FI observed in birds fed WTM diets at starter phase. According to Rath et al. (2000) high bone weight and density indicates good bone health in birds.

The reduced TBW and TBD of birds fed WTM5 and WT10 than control group at grower phase may be as a result of high fat contents present in WTM (Table 1) which may have interfered with the normal bone growth and metabolism in birds. Similar findings were observed by Sumbule et al. (2021) and Rytlewski et al., (2025). However, the results disagree with findings by (Sarıca et al., 2025), who supplemented 0.4% insect meal as feed additive in broiler diets.

Better BBS observed in birds on WTM5 compared to other treatment groups at grower phase may be associated with high ash contents observed in WTM (Table 1) as well as increased TBW and TBD observed at starter phase. According to Shim et al. (2012), higher tibia BBS aid in withstanding gravity while carrying the additional weight of broiler chickens. However, in contrast, Uushona (2015) observed non-significant differences on BBS of broiler chickens when replacing SBM with black soldier fly as a protein source throughout the growing period. This contradiction may be due to the use of different chicken strain, insect species and inclusion levels. Although it was expected that WTM10 perform better than WTM5 based on inclusion levels, however, that was not the case since chitin toxicity levels elevate with increase in WTM in diets. Thus, suggesting that WTM inclusion above 5% could negatively affects BBS of broiler chickens.

## Conclusion

WTM inclusion affected FI, WBC, lymphocytes, %PCV, H/L ratio, ALT, AST, bilirubin, TBW, TBD, and BBS without any health risks of broiler chickens. Thus, it could be concluded that WTM of up to 10% could be supplemented in broiler chicken diets as an alternative protein feed ingredient without adverse effects on the performance, blood and bone parameters of broiler chickens from 1 to 42 days. Further research trials on digestibility, protein intake and efficiency ratio of WTM as a partial substitute for SBM in broiler chicken diets is recommended.

## Declaration of AI and AI-assisted technologies

Authors declare that no AI or AI-assisted Technologies used in compilation of this manuscript.

## Ethics approval and consent to participate

This study was approved by the Animal Ethics Committee of the College of Agriculture and Environmental Science at the University of South Africa, with approval number 2022/CAES_AREC/150.

## Funding

This project was funded by the National Skills Fund (NSF) under the National research foundation (NRF) Scarce Skills funding scholarship opportunity (Ref. MND210402591791) as well as the Insect Project (ASDG-RSP).

## CRediT authorship contribution statement

**Sekobane Daniel Kolobe:** Writing – review & editing, Writing – original draft, Visualization, Software, Resources, Methodology, Formal analysis, Data curation. **Amenda Nthabiseng Sebola:** Writing – review & editing, Supervision, Conceptualization. **Emmanuel Malematja:** Validation, Software, Investigation, Formal analysis. **Mabelebele Monnye:** Writing – review & editing, Visualization, Validation, Supervision, Project administration, Funding acquisition, Conceptualization.

## Disclosures

All the authors do not have any conflicts of interest to declare.
